# Does Appearance Matter during Pregnancy? A Cross-Sectional Study of Body Satisfaction from Pre-Pregnancy to Late Gestation

**DOI:** 10.3390/ijerph192316375

**Published:** 2022-12-06

**Authors:** Emilie Mass Dalhaug, Lene Annette Hagen Haakstad

**Affiliations:** Department of Sports Medicine, Norwegian School of Sport Sciences, 0863 Oslo, Norway

**Keywords:** body judgement, body satisfaction, gestational weight gain, physical activity, pregnancy

## Abstract

Few studies have explored the associations between body satisfaction and physical activity and weight gain during pregnancy, and none have been conducted in Scandinavia. Hence, the aim of the present study was to evaluate changes in body satisfaction from pre-pregnancy to late pregnancy and investigate whether this differed according to parity. We also wanted to explore the association between body satisfaction and physical activity and weight gain among pregnant women in Norway. This cross-sectional survey used an electronic questionnaire to assess physical activity level, weight gain and women’s satisfaction with body weight and size. In total, 150 pregnant women answered the questionnaire. Related-samples Wilcoxon signed rank tests, Mann–Whitney U tests and chi-square tests were used to answer our research questions. The proportion of women who were dissatisfied with their body weight and shape increased from pre-pregnancy to late gestation (body weight *p* = 0.030 and body shape *p* = 0.040). Body dissatisfaction before and during pregnancy was linked to weight gain above recommendations. Characterising oneself as physically active prior to pregnancy was associated with satisfaction with body shape pre-pregnancy. Given that mothers strongly influence how a child will judge their body later in life, the results of this study underline the importance of addressing these issues during pregnancy.

## 1. Introduction

Body dissatisfaction is the degree to which individuals experience the discrepancy between their cognitive and ideal body weight and shape [1]. This can be partly attributed to the existence of societal and media pressure to be thin [2,3]. Given that a woman’s body weight and size change rapidly and profoundly during pregnancy, women who retain societal standards of appearance are likely to experience increased body dissatisfaction [4]. Although women understand the need for gaining weight while pregnant [5], research examining body dissatisfaction during pregnancy has found a wide variation in women’s reactions to these physical changes, ranging from increased to stable [4,6], to decreased body satisfaction [3,7]. Yet, results from studies suggest that, regardless of these physical changes, pregnant women have higher body satisfaction than their non-pregnant peers [6]. Studies also suggest that multiparous women feel more positive about changes to their body during pregnancy, compared with nulliparous women [3].

It is well-established that meeting the recommended levels of physical activity [8] and gaining weight within the Institute of Medicine guidelines [9] may lower the incidence and severity of serious conditions associated with pregnancy, including gestational diabetes mellitus [10,11], pregnancy-induced hypertension [11], preterm birth [12], macrosomia [10,12], and small for gestational age infants [12]. Attempts have been made, mainly in Australia and the US, to evaluate the association between body satisfaction and health behaviours, including physical activity [13,14] and weight gain outside recommended levels [7,15]. A systematic review and meta-analysis of cohort studies [13] showed that physical activity was positively associated with body satisfaction among pregnant women. With regard to weight gain during pregnancy, the main findings are that women who report being dissatisfied with their body pre-pregnancy are more likely to gain weight above recommended levels [7,15]. Additionally, a thinner body size preference has been associated with excessive gestational weight gain [15].

Understanding how being pregnant may affect body satisfaction should receive close attention, since women judging their bodies negatively are more prone to antenatal and postpartum depression [16], anxiety [17], and inadequate or restrictive diet [4,18]. In addition, maternal body dissatisfaction should be viewed from a cultural perspective, and absent from the literature is knowledge of how Scandinavian women judge their bodies during pregnancy, and how this is linked to physical activity (PA) and gestational weight gain (GWG). Therefore, we aim to evaluate changes in body satisfaction from pre-pregnancy to late pregnancy and investigate whether this differs according to parity. We also want to explore the possible association between body satisfaction and health behaviour, including PA and GWG, among pregnant women in Norway.

## 2. Materials and Methods

### 2.1. Study Design

This investigation was part of a larger cross-sectional survey on health behaviours and information sources among pregnant women in Oslo, Norway [19]. The study was reviewed by the Regional Committee for Medical and Health Research Ethics (REK 2015/1941 A), who concluded that, according to the act on medical and health research (the Health Research Act 2008), the study did not require full review by REK. The study was approved by the Norwegian Social Science Data Service (NSD 45111), and conducted in accordance with the Declaration of Helsinki.

### 2.2. Participants

Enrolment was limited to women living in Oslo, ≥18 years, ≥20 weeks gestation, and able to read and write Norwegian. Women not living in Oslo were excluded from the analysis. The questionnaire was administered only once for each woman during their pregnancy.

In an endeavour to ensure a representative sample with regard to different age groups, socioeconomic backgrounds and ethnicities, all antenatal clinics in Oslo (*n* = 18), both urban and rural, were invited to participate in this project. However, 16 antenatal clinics declined participation, due to other ongoing research projects. Consequently, we chose to recruit participants through advertisements on Facebook and Instagram, as well as through various pregnancy-related online chat forums and the university website. The advertisement on Facebook and Instagram was not limited to pregnant women, but targeted all women living in Oslo. The internet-based questionnaire was active during June to August 2016.

### 2.3. Outcome Measures

The multidimensional electronic survey contained 101 questions and was developed using existing and validated questions [20,21,22,23], as well as some newly developed questions suitable to the purpose of this study. The current analysis focused on changes in body satisfaction from pre-pregnancy (assessed retrospectively) to current pregnancy week (range 20–42) and whether this was related to various health behaviours, including PA, GWG and behavioural changes to stabilize/reduce GWG during pregnancy. Questions were a mix of 11-point Likert scales, close-ended questions, and semi-close-ended questions with the option to elaborate (Table 1). The questionnaire was piloted for comprehensibility of questions and answer options among 23 pregnant women and was revised accordingly. A full questionnaire in Norwegian may be provided upon request.

### 2.4. Statistical Analyses

All statistical analyses were conducted using IBM SPSS Statistical Software version 28.0 for Windows. Background variables are presented as frequencies, percentages, or means with standard deviation. Whether a woman had gained weight below, within, or above the GWG guidelines was calculated using mean recommended weight gain in the first trimester (1.5 kg), adding the mean recommended number of grams per week multiplied by the number of weeks the woman was pregnant above the first trimester [27]. Pre-pregnancy BMI was used to determine weight gain below, within, or above the GWG guidelines. Dissatisfaction and satisfaction with body weight and shape was defined as a score ≤3 and ≥7 on an 11-point scale, respectively [28]. Changes in body satisfaction was evaluated using related-samples Wilcoxon signed rank test. Differences in body satisfaction between groups was calculated with Mann–Whitney U tests. The relationship between body satisfaction and health behaviours was assessed by chi-square tests and Fisher’s exact tests.

## 3. Results

### 3.1. Participants

Participant characteristics are shown in Table 2. Responses were received from 275 pregnant women, 244 recruited through social media and 31 recruited through antenatal clinics. All analyses included data from the 150 participants who fully completed the questionnaire and provided information on body satisfaction and health behaviours. Age ranged from 19 to 45 with a mean of 31.1 (±4.3) years. Mean gestation week was 30.6 (±5.9) and mean pre-pregnancy BMI was 24.2 (±4.2) kg/m^2^.

### 3.2. Health Status and Behaviour

Almost 90% of women reported that they were physically active for a minimum of 150 min of moderate intensity each week pre-pregnancy. This number halved in current gestation week (Table 2).

As shown in Table 3, nearly 65% of the women had gained weight outside the GWG guidelines. Forty-seven women (31.3%) had made habitual changes in order to stabilise/reduce further weight gain, with “*Deliberately omitted foods high in sugar and fat*” (*n* = 37, 24.7%) being the most frequently reported change, followed by “*Increased the number of exercise sessions*” (*n* = 7, 4.7%) and “*Eaten less than usual*” (*n* = 7, 4.7%).

The majority of women (60.7%) rated their physical health as good or very good. This was also evident for mental health (74.7%).

### 3.3. Changes in Body Satisfaction from Pre-Pregnancy to Late Pregnancy

**Figure 1:**
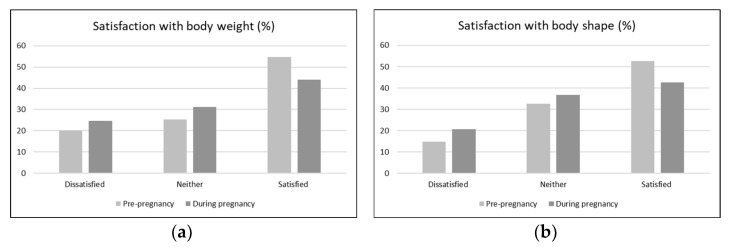
Changes (%) in satisfaction with (**a**) body weight and (**b**) body shape from pre-pregnancy to during pregnancy.

Most women were satisfied with their body weight and shape both prior to and during pregnancy. Still, 20% of women reported to be dissatisfied with their body weight pre-pregnancy, while 14.7% reported that they were dissatisfied with their body shape. This increased to 24.7% and 20.7% during pregnancy, respectively (Figure 1). Simultaneously, the number of women who were satisfied with their body weight (*n* = 82, 54.7%) and body shape (*n* = 79, 52.7%) pre-pregnancy decreased to 66 (44%) and 64 (42.7%), respectively, during pregnancy (Figure 1). The decrease in body satisfaction from pre-pregnancy to during pregnancy was significant for body weight (*p* = 0.03) and body shape (*p* = 0.04).

When comparing the means between nulliparous and multiparous women, nulliparous women were more satisfied with their body weight (*p* = 0.008) and body shape (*p* = 0.003) pre-pregnancy. However, we found no difference between the groups during pregnancy (*p* = 0.23 and *p* = 0.27 for body weight and body shape, respectively). In the multiparous group, satisfaction with body weight remained completely stable from pre-pregnancy to late gestation (mean 5.31 to 5.31) and did only slightly increase for body shape (mean 5.31 to 5.51).

### 3.4. Associations between Body Satisfaction and Health Behaviour

#### 3.4.1. Physical Activity

We found an association between characterising oneself as physically active pre-pregnancy and satisfaction with body shape pre-pregnancy (*p* = 0.02). Otherwise, we found no associations between characterising oneself as physically active prior to or during pregnancy and satisfaction with body weight pre-pregnancy or body weight and shape during pregnancy.

#### 3.4.2. Gestational Weight Gain

**Figure 2:**
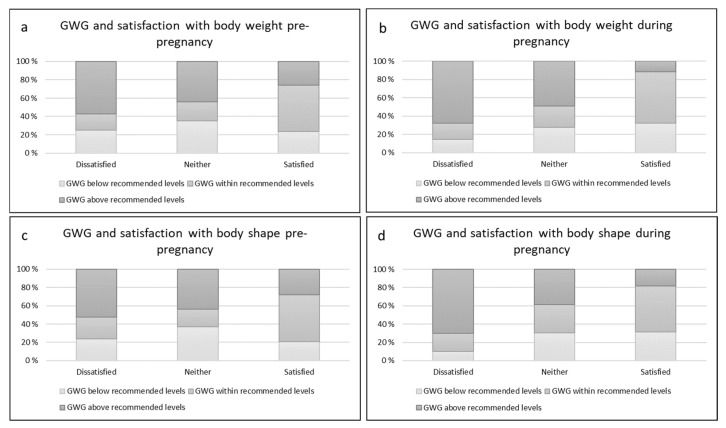
Distribution (%) of those gaining below, within or above the recommended levels in association to degree of satisfaction with (**a**) body weight pre-pregnancy, (**b**) body weight during pregnancy, (**c**) body shape pre-pregnancy and (**d**) body shape during pregnancy.

Being dissatisfied with body weight and shape prior to and during pregnancy was linked to GWG above recommended levels (Figure 2). We also found that satisfaction with body weight and shape, both prior to and during pregnancy, was associated with gaining within recommended levels (Figure 2).

## 4. Discussion

The aim of this study was to examine changes in body satisfaction from pre-pregnancy to late gestation and explore whether body satisfaction was associated with physical activity and weight gain among Norwegian pregnant women. In general, more women were satisfied than dissatisfied with their body weight and shape both before and during pregnancy. The proportion of women who were dissatisfied with their body weight and shape increased from pre-pregnancy to late gestation, while the number of women who were satisfied with their body weight and shape decreased. Further, we found a decrease in body satisfaction among nulliparous women, while body satisfaction remained stable in the multiparous group. Body dissatisfaction was associated with GWG above recommended levels, and characterising oneself as physically active pre-pregnancy was associated with satisfaction with body shape pre-pregnancy.

Previous research, mainly from Australia and the US, both support [3,7,29,30] and contradict [4,6] our findings. Loth and colleagues [6] found that women experienced improved body satisfaction during pregnancy, despite changes in body shape and size. Duncombe and colleagues [4] found that body satisfaction was relatively stable across pregnancy. Others have found that body dissatisfaction during pregnancy was common [3] and that body satisfaction decreased during pregnancy [29,30]. These inconsistencies in results may be related to differences in utilised methodologies (e.g., cross-sectional and longitudinal design), time of assessment (e.g., early or late pregnancy), procedures and the populations studied [6,31]. On the other hand, it may also reflect the complexity of measuring body satisfaction. As evident in the literature, body satisfaction is a multi-dimensional construct of various aspects (e.g., salience of weight and shape, perceptions regarding what size and shape is ‘ideal’ and perceptions of being strong and fit [1]). Studies reporting stability of satisfaction [4] and studies reporting decreased satisfaction [3,30] have assessed different dimensions of body satisfaction, indicating that body satisfaction may change depending on the dimensions assessed.

Our results suggest that multiparous women are more mentally robust against bodily changes during pregnancy. In this group, body satisfaction remained completely stable, while it decreased among nulliparous women. This is in line with Hicks and Brown [3] showing that the more children a mother had, the more positive she felt about changes to her body.

We found that characterising oneself as physically active prior to pregnancy was associated with satisfaction with body shape pre-pregnancy. No other associations between PA and body satisfaction were found. This was surprising, as previous research has shown that regular exercise is positively related to body satisfaction in pregnant women [32,33,34]. One possible explanation is that we assessed PA by asking whether they characterised themselves as physically active according to the health authorities’ recommendations of exercise during pregnancy. If we had assessed different dimensions of PA (type, frequency, intensity and duration of activity), the results might have been different. Additionally, prefacing the question with the recommendations from the health authorities may lead to social desirability bias, whereby women are more likely to say they meet physical activity guidelines when they do not. This may be the reason why almost 90% of women in our sample reported that they were physically active prior to pregnancy. Further, the recommendations that was quoted is specific to pregnant women, which requires women to infer whether the same recommendations apply prior to pregnancy as well. This may have led women to rate their pre-pregnancy PA level without a clear description of the actual recommendations on PA. Because of the small number of women not characterising themselves as physically active prior to pregnancy, the analyses on pre-pregnancy physical activity were of low statistical power.

Consistent with the findings of Bagheri and colleagues [35], the vast majority of women gaining weight within the IOM recommendations were satisfied with their body weight and shape. We also found that being dissatisfied with own body weight and shape prior to and during pregnancy was linked to GWG above recommended levels, corresponding to the results from a systematic review [15]. A recent analysis of data from 4429 women concluded that adhering to the IOM recommendations is difficult, with two-thirds gaining weight outside recommended levels [36]. Together with the well-documented negative effects excessive GWG has on various pregnancy outcomes [36], this underlines the importance of effective programs for preventing excessive GWG. A recent systematic review and meta-analysis of 117 randomised clinical trials (involving 34,546 pregnancies), found that antenatal diet and physical activity-based lifestyle interventions were associated with a small reduction in mean GWG (−1.13 kg) [37]. While such interventions have been shown to be slightly effective, our findings suggest that pregnancy programs with the aim to reduce excessive GWG should also include strategies to improve body satisfaction, and ideally be implemented when women are planning to become pregnant.

Several studies have found an association between media use and body judgement during pregnancy [3,38,39]. Hicks and Brown [3] found that time spent on social media was associated with body dissatisfaction among pregnant women. Further, women have stated that they felt that the media’s depiction of pregnancy was unrealistic, and nearly half the women reported feeling negative about their body due to pregnant media images [38]. This is in line with body dissatisfaction research, both in pregnant and non-pregnant populations, showing that exposure to unrealistic beauty ideals in social media may lead to appearance comparisons, internalisations, self-objectification, and body monitoring [38,40]. Although body dissatisfaction was not associated with choosing internet and media as the most important sources of pregnancy information in this sample [19], the majority of women were recruited to participate in the study through social media platforms, which may have impacted the current results.

### 4.1. Strengths, Limitations, and Future Directions

To our knowledge, this is the first study to investigate body satisfaction and possible associations with PA and GWG in a pregnant Scandinavian population. Hence, our results are bridging the gap regarding possible cultural differences. We used an electronic questionnaire, with questions based on previously validated instruments and questions used in similar studies [20,21,22,23]. We also included questions on socioeconomic status, marital status, ethnicity, physical and mental health, which could potentially impact body satisfaction [41]. Additionally, online surveys are time efficient and cost-effective [42]. Further, we made it clear to all participants that we were not collecting any identifying information (like names and addresses). Responses could therefore not be traced back. Thus, unlike interviews, questionnaires are good for examining more sensitive topics, with participants being more honest when they are not asked in person [43].

Still, some issues must be considered when interpreting our findings. First, most women in our study were married, highly active, highly educated, Nordic Caucasian and living in the capital city of Norway. Women living in urban areas often have higher socioeconomic status and may be more prone to follow guidelines than women with lower socioeconomic status [44]. This may limit the generalisability of our findings to other pregnant populations due to potential selection bias. Second, participants answered the questionnaire during pregnancy week 20 to 42. This large range in time of assessment could potentially have impacted participant’s ratings of body satisfaction. However, subgroup analyses showed no difference on the questions regarding body satisfaction between those answering in week 20–25 and 36–42 (*p* = 0.6 for body weight and *p* = 0.8 for body shape). Nevertheless, future studies should choose a narrower range in pregnancy week to ease comparison with other studies. Third, when our initial approach of recruiting women through all 18 antenatal clinics in Oslo failed, we chose to recruit through social media. We are aware that this may have impacted the results. Others have, however, concluded that recruitment through social media is an effective and efficient way to recruit participants, especially in harder-to-reach populations [45]. Fourth, all information was self-reported and therefore subjective to social desirability bias. Finally, because of the cross-sectional nature of our design, questions on pre-pregnancy body satisfaction were answered retrospectively. More research is needed to replicate our findings in a more diverse sample of pregnant women. Additionally, future studies should rely on a longitudinal design with follow-up measures at several time points both before and during pregnancy.

### 4.2. Implications for Professional Practice

Although most women were satisfied with their body before and during pregnancy, the number of women who felt dissatisfied increased from pre-pregnancy to late gestation. Our study also found that dissatisfaction with own body was associated with GWG above recommended levels. Given the negative health effects GWG above guidelines has on both mother and child [36], health professionals should communicate with and ask their patients important questions about body satisfaction during early pregnancy, and take action if the woman shows signs of dissatisfaction. As research has shown a modest reduction in GWG from PA and diet interventions [37], the inclusion of strategies to improve body satisfaction could possibly make a positive impact on GWG.

## 5. Conclusions

In conclusion, the proportion of women being dissatisfied with their body weight and shape increased from pre-pregnancy to late gestation. We also found that body satisfaction was associated with GWG above recommended levels and characterising oneself as physically active pre-pregnancy was associated with satisfaction with body shape pre-pregnancy. Since mothers strongly influence how their child will judge their own body later in life [46], the results of this study underline the importance of addressing these issues during pregnancy.

## Figures and Tables

**Table 1:** Dimensions assessed and main variables and questions used to answer the research questions.

Dimensions Assessed	Main Variables and Questions Used	Reference
Sociodemographic characteristics	Age, gestation week, parity, marital status, place of residence, country of birth, educational level, occupation, and number of antenatal consultations.	Developed for this project
Anthropometry and gestational weight gain	Participants were asked to state their height, pre-pregnancy weight, and current weight ^a^.	Developed for this project
Physical activity	Assessed using the question: “The health authorities recommend all pregnant women to perform moderate-intensity aerobic physical activity (activities that take moderate physical effort and make you breathe somewhat harder than normal, such as brisk walking, housework, etc.) for a minimum of 30 min five days a week. With this in mind, would you characterize yourself as physically active (a) pre-pregnancy and (b) in your current gestation week?” Response options: “*Yes*”, “*No*” or “*I don’t know*”.	Based on the ACOG recommendations [7]
Changes in body satisfaction from pre-pregnancy to late pregnancy	The respondents were asked to rate four statements on an 11-item scale, 0 being negative and 10 being positive. The statements were: (1) “*How satisfied were you with your body weight pre-pregnancy?*”, (2) “*How satisfied are you with your body weight today?*”, (3) “*How satisfied were you with your body shape pre-pregnancy?*” and (4) “*How satisfied are you with your body shape today?*”.	Based on questions used in previous research [24,25]
Habitual changes to stabilise/reduce weight gain during pregnancy.	Assessed using the question: “Over the course of pregnancy have you made habitual changes in order to stabilise/reduce further weight gain?”. Response options: “*Yes*” or “*No*”. If the respondents answered yes, they were asked to elaborate. Categorical response options: “*Increased the number of exercise sessions*”, “*Increased the exercise intensity*”, “*Skipped breakfast*”, “*Deliberately omitted foods high in sugar and fat*”, “*Eaten less than usual*” and “*Other, please specify*”. The respondents were able to choose more than one category.	Developed for this project
Satisfaction with physical and mental health	The respondents were asked to rate four statements on an 11-item scale, 0 being negative and 10 being positive. The statements were: “*All in all, how satisfied are you with your physical health as pregnant?*” and “*All in all, how satisfied are you with your mental health as pregnant?*”.	Developed for this project

^a^ Pre-pregnancy height and weight were used to calculate pre-pregnancy BMI. BMI categories and GWG ranges were consistent with the World Health Organization’s (WHO) guidelines [26] and the guidelines from the IOM [27]. Pre-pregnancy weight and current weight were used to calculate GWG.

**Table 2:** Participant characteristics (*n* = 150).

Characteristics		*n*	%
Parity			
	Nulliparous	91	60.7
	Multiparous	59	39.3
Marital status			
	Married/living together	147	98.0
	Other	3	2.0
Country of birth			
	Norway	130	86.7
	Other (Sweden, Denmark, Iceland, Syria, Gambia, Macedonia, Morocco, Spain, Italy, Russia, Iran)	20	13.3
Education			
	<4 years college/university	54	36.0
	≥4 years college/university	96	64.0
Employment status			
	Employed/student	144	96.0
	Not employed	6	4.0
Physically active			
	Pre-pregnancy	132	88.0
	During pregnancy	73	48.7
Pre-pregnancy BMI category			
	Underweight	2	1.3
	Normal weight	102	68.4
	Overweight	28	18.7
	Obese	17	11.4
Smoking in pregnancy			
	No	149	99.3
	Yes	1	0.7
Pregnancy complaints			
	Pelvic girdle pain	69	46.0
	Back pain	67	44.7
	Urinary incontinence	30	20.0
On sick leave		39	26.0
Adherence to national nutritional guidelines		98	65.3

**Table 3:** Women gaining within, below or above the IOM recommendations (*n* = 139). Data are presented in frequency (n), percentage (%) and mean kg (SD) below and above recommendations.

	n	%	Mean (SD)
Within recommendations	51	36.7	-
Below recommendations	37	26.7	−2.6 (± 2.2)
Above recommendations	51	36.7	+3.0 (± 2.4)

## Data Availability

A full questionnaire in Norwegian may be provided upon request.
